# High-quality imaging of endolymphatic hydrops acquired in 7 minutes using sensitive hT_2_W–3D–FLAIR reconstructed with magnitude and zero-filled interpolation

**DOI:** 10.1007/s00405-021-06912-4

**Published:** 2021-06-18

**Authors:** Jing Zou, Luguang Chen, Hongbin Li, Guoping Zhang, Ilmari Pyykkö, Jianping Lu

**Affiliations:** 1 Department of Otolaryngology-Head and Neck Surgery, Center for Otolaryngology-Head and Neck Surgery of Chinese PLA, Changhai Hospital, Second Military Medical University, Shanghai, China; 2 Department of Radiology, Changhai Hospital, Second Military Medical University, Shanghai, China; 3 Hearing and Balance Research Unit, Field of Otolaryngology, School of Medicine, Faculty of Medicine and Health Technology, Tampere University, Tampere, Finland

**Keywords:** Meniere’s disease, Endolymphatic hydrops, MRI, Image reconstruction, Enhancement

## Abstract

**Background:**

It is still challenging to detect endolymphatic hydrops (EH) in patients with Meniere’s disease (MD) using MRI. The aim of the present study was to optimize a sensitive technique generating strong contrast enhancement from minimum gadolinium–diethylenetriamine pentaacetic acid (Gd–DTPA) while reliably detecting EH in the inner ear, including the apex.

**Materials and methods:**

All imaging was performed using a 3.0 T MR system 24 h after intratympanic injection of low-dose Gd–DTPA. Heavily T2-weighted 3-dimensional fluid-attenuated inversion recovery reconstructed with magnitude and zero-filled interpolation (hT_2_W–FLAIR–ZFI) was optimized and validated in phantom studies and compared with medium inversion time inversion recovery imaging with magnitude reconstruction (MIIRMR). The following parameters were used in hT_2_W–FLAIR–ZFI: repetition time 14,000 ms, echo time 663 ms, inversion time 2900 ms, flip angle 120°, echo train length 271, and field of view 166 × 196 mm^2^.

**Results:**

MRI obtained using hT_2_W–FLAIR–MZFI yielded high-quality images with sharper and smoother borders between the endolymph and perilymph and a higher signal intensity ratio and more homogenous perilymph enhancement than those generated with MIIRMR (*p* < 0.01). There were predominantly grade II EHs in the cochleae and grade III EHs in the vestibule in definite MD. EH was detected in the apex of 11/16 ipsilateral ears, 3/16 contralateral ears in unilateral definite MD and 3/6 ears in bilateral MD.

**Conclusions:**

The novel hT_2_W–FLAIR–MZFI technique is sensitive and demonstrates strong and homogenous enhancement by minimum Gd–DTPA in the inner ear, including the apex, and yields high-quality images with sharp borders between the endolymph and perilymph.

**Supplementary Information:**

The online version contains supplementary material available at 10.1007/s00405-021-06912-4.

## Introduction

MRI has been increasingly used to assess endolymphatic hydrops (EH) in clinical practice to diagnose inner ear diseases, especially Meniere’s disease (MD), since the first report of an animal study in 2000 and following optimization of the MRI protocol [1–6]. Strong contrast enhancement of the perilymph is essential for reporting reliable results of EH, which depends on sufficient distribution of the contrast agents (gadolinium chelate) in the inner ear and the MRI sequence. However, this is not guaranteed with the current technique. Although the barrier from blood to perilymph is looser than that to endolymph, entry of contrast agents into the perilymph after intravenous injection of gadolinium chelate is still hindered by the rather tight blood-perilymph barrier unless it is severely impaired [7–9]. Aiming to boost the uptake of contrast agents in the inner ear, intratympanic administration of gadolinium chelate was developed as an alternative method for imaging EH in the clinic, but this method still failed in 13% of patients [3, 10, 11]. Uneven diffusion of contrast agents inside the inner ear resulted in weak distribution of gadolinium chelate in the cochlear apex regardless of whether the contrast agents were delivered intratympanically or intravenously and may also affect the reliability of the method in the detection of EH because hydrops is suspected to start from the apex in patients with low-tone hearing loss [12–14]. In animal experiments with a two-phase EH model, a combination of chronic endolymphatic sac dysfunction and acute stress-induced endolymph production, the most severe degree of EH was present in the cochlear apex [15]. Therefore, there is a need in the clinic to develop a more sensitive method for the detection of EH.

Naganawa et al. aimed to improve the sensitivity of MRI methods for the detection of EH and reported that heavily T_2_-weighted three-dimensional fluid-attenuated inversion recovery (hT_2_W–3D–FLAIR) and a hybrid of reversed images of positive endolymph signals and native images of perilymph signals multiplied with heavily T_2_-weighted MR cisternography (HYDROPS-Mi2) imaging were optimized methods [7, 16]. The group recently further optimized the parameters of 3D-real inversion recovery imaging based on phase-sensitive reconstruction by prolonging the repetition time and increasing the refocusing flip angle, which yielded a similar value in detecting EH to HYDROPS-Mi2 imaging, which is complicated and time consuming for clinical applications [14, 17]. Zou et al. further improved the contrast enhancement using magnitude reconstruction, which was named medium inversion time inversion recovery imaging with magnitude reconstruction (MIIRMR), and demonstrated a sharper border between the perilymph and the endolymph than that when using hT2W–3D–FLAIR after intratympanic injection of 20-fold-diluted gadolinium–diethylenetriamine pentaacetic acid (Gd–DTPA) [18]. During a pilot study, magnitude reconstruction of MIIRMR was compared with real reconstruction of 3D-real inversion recovery imaging as recently reported using otherwise identical parameters (Table 1) [17]. MIIRMR was significantly more sensitive than 3D-real inversion recovery (Supplementary information 1). The scan time of MIIRMR was significantly shorter than that of 3D-real inversion recovery imaging (8.6 min versus 11.4 min), although a coil with fewer channels was used (20 versus 32 channels) [17, 18]. However, the enhancement in the perilymph of the cochlear apex was still weak in most cases. Zero-filled interpolation (ZFI) is a technique used to reduce partial-volume artifacts and to improve the appearance of small structures and edges while retaining genuine information of the raw images [19]. ZFI has been applied in intracranial MR angiography to improve the contrast and continuity of examined small vessels in the range of 0.2–0.6 mm but leads to insignificant improvement in the accuracy of luminal measurement in medium-sized vessels [20, 21]. We hypothesized that T_2_W–3D–FLAIR reconstructed with magnitude and ZFI (hT_2_W–FLAIR–MZFI) may further improve the demonstration of EH in gadolinium enhancement MRI, especially in the cochlear apex, which has a rather small size within the above-mentioned range and a usually weak uptake of gadolinium chelate [22, 23].

**Table 1:** The parameters for hT2W–FLAIR–MZFI, MIIRMR and 3D-real inversion recovery sequences

Sequences	TR/TE (ms)	TI (ms)	FA	ETL	Matrix		FOV (mm)	Rec	Time (min)
					scan	Rec			
hT_2_W–FLAIR–MZFI^a^	14,000/663	900	120°	271	324 × 384	648 × 768	166 × 196	Mag + ZFI	7.4
hT_2_W–FLAIR–MZFI^b^	7000–16,000/663	2900	120°	271	324 × 384	648 × 768	166 × 196	Mag + ZFI	3.3–8.8
MIIRMR	16,000/663	2700	120°	271	324 × 384	324 × 384	166 × 196	Mag	8.8
3D real IR	16,000/663	2700	120°	271	324 × 384	324 × 384	166 × 196	Real	8.8

*3D real IR* 3D-real inversion recovery, *ETL* echo chain length, *FA*: flip angle, *FOV* field of view; hT_2_W–FLAIR–MZFI^a^: heavily T_2_-weighted 3D-fluid attenuated inversion recovery reconstructed with magnitude and zero-filled interpolation that was applied in patient studies; hT_2_W–FLAIR–MZFI^b^: hT_2_W–FLAIR–MZFI used in phantom studies for comparisons of TR-dependent sensitivity with the following *TR* 16,000 ms, 14,000 ms, 12,000 ms, 9000 ms, and 7000 ms, *MIIRMR* medium inversion time inversion recovery imaging with magnitude reconstruction, *Rec* reconstruction, *TE* echo time, *TI* inversion time, *Time* scan time, *TR* repetition time, *Mag* magnitude reconstruction. Other parameters: fat saturation; GRAPPA: 2; Flip angle mode: constant; number of excitations: 2; slice partial fourier: 6/8; slices per slab: 30 in hT2W–FLAIR–ZFI^b^ and 60 in other sequences; slice thickness: 1 mm; voxel size = 0.26 × 0.26 × 1.0 mm^3^ for hT_2_W–FLAIR–MZFI^a^ and hT_2_W–FLAIR–ZFI^b^, and 0.52 × 0.52 × 1.0 mm^3^ for MIIRMR and 3D real IR

The purpose of the present work was to demonstrate a technique employing hT_2_W–FLAIR–MZFI that was capable of generating strong contrast enhancement from minimum Gd–DTPA to reliably detect EH even in the cochlear apex of patients and to compare the results with those derived using the MIIRMR method.

## Materials and methods

### Experimental optimization of the imaging method

#### Empirical study

Five patients diagnosed with definite MD (for the inclusion criteria, see section (“Clinical evaluation of the developed imaging method”) were selected for the empirical MRI studies with respect to the reconstruction method, parameter optimization of hT_2_W–FLAIR–MZFI imaging, and dilution of Gd–DTPA. MRI was performed 24 h postinjection of Gd–DTPA using a 3 T MR system (MAGNETOM Skyra, Siemens Healthcare, Erlangen, Germany) equipped with a 20-channel head/neck coil. After testing with various repetition time (TR) and inversion time (TI) values, TR 14,000 ms and TI 2900 ms were chosen (Table 1). Various dilutions of Gd–DTPA (9.38 g, 20 ml, Beijing Beilu Pharmaceutical CO., LTD, Beijing, China) (20-, 25-, and 30-fold) were tested in four patients with the diseased ear fixed at a 20-fold dilution, while the contralateral ear was exposed to a lower concentration following a previously reported protocol of intratympanic injection of 0.1 ml of the above diluted Gd–DTPA that was directly delivered onto the posterior upper surface of the tympanic medial wall using a catheter passing through the perforated tympanic membrane [18, 24, 25]. Finally, a 20-fold dilution, which was also reported to induce sufficient uptake of Gd–DTPA in the inner ear with the least individual variability [25], was defined as the optimized concentration in the following clinical study.

#### Phantom study

Three serial dilutions of Gd–DTPA were prepared and injected into 2-ml plastic cryopreservation tubes as follows. The first sample (phantom 1) was serially diluted from 1 × 10^3^-fold through 1 × 10^8^-fold with an interval of tenfold using physiological saline. Since the perilymph contains proteins at approximately 2.8 mg/ml and the perilymph/cerebrospinal fluid (CSF) protein ratios are 5 in humans and 12.5 in guinea pigs [26], bovine serum albumin (BSA) samples were prepared using physiological saline at concentrations of 11.2, 5.6, and 2.8 mg/ml to test the potential contribution of perilymphatic proteins to signal production. Based on the results of the first study, the second sample (phantom 2) was serially diluted from 2 × 10^3^ to 5.12 × 10^5^. To assess the impact of repetition time on the sensitivity of hT2W–FLAIR–MZFI, the third sample (phantom 3) was serially diluted from 2 × 10^3^ to 1.28 × 10^5^. Tubes containing physiological saline were used as blank controls, and duplicates were prepared for each dilution. Phantoms 1 and 2 were imaged using both hT_2_W–FLAIR–MZFI and MIIRMR sequences (Table 1). Phantom 3 was imaged using the hT_2_W–FLAIR–MZFI sequence with the following TRs: 16,000, 14,000, 12,000, 9000, and 7000 ms (Table 1).

### Clinical evaluation of the developed imaging method

#### Patient information

Patients who visited author JZ at the Department of Otolaryngology-Head & Neck Surgery, Changhai Hospital, Second Military Medical University, from June to November 2020 were screened. The inclusion criteria were as follows: (1) diagnosis of definite or probable MD made according to the criteria of the Barany Society in 2015 [27] and (2) sudden sensorineural hearing loss accompanied by vertigo or constant balance problems meeting the disease definitions (AAO-HNS, 2012) [28]. The exclusion criteria were as follows: (1) vertigo with other origins, such as benign paroxysmal positional vertigo and diseases in the central nervous system; and (2) sensorineural hearing loss without vertigo. In total, 22 patients diagnosed with MD (aged 28–74 years, average age 47.1 years; 10 males and 12 females) and one patient diagnosed with sudden sensorineural hearing loss (79-years old, female) were recruited for the study. The participants formed a consecutive series.

#### Imaging

Each patient was imaged 24 h after intratympanic injection of 0.1 ml of 20-fold-diluted Gd–DTPA that was delivered onto the posterior upper part of the tympanic medial wall as previously reported [18, 24, 25]. In total, one patient was imaged with hT_2_W–FLAIR–MZFI, MIIRMR and 3D-real inversion recovery, 10 patients were imaged using both hT_2_W-FLAIR-MZFI and MIIRMR, and 12 patients were imaged only with hT_2_W–FLAIR–MZFI (Supplementary information 2). Parameters for hT_2_W–FLAIR–MZFI and MIIRMR are shown in Table 1. T_2_-weighted-sampling perfection with application-optimized contrasts using a flip angle evolution (T_2_W-SPACE) sequence was applied in all cases to image potential inner ear fibrosis or vestibular schwannoma, as previously reported [18].

#### Image analysis

All images were sent to an advanced workstation (Leonardo, Siemens Healthcare, Germany) for further analysis by author JZ, who has more than 21 years of experience in inner ear imaging [1, 2]. The maximum cross sectional slices of the cochlea and vestibule were chosen for image analysis. First, the general image quality with respect to the border between the unenhanced endolymph and enhanced perilymph was subjectively evaluated. Image sensitivity, diversity of the inner ear enhancement signal, and sensitivity in detecting EH in the cochlea, including the apex, were assessed. The sensitivity was expressed as signal intensity ratios (SIRs) in various regions of interest (ROIs) by manual drawing. The diversity of signal intensity was displayed as differences in SIR between different sites of the inner ear. Finally, the sensitivity was tested by analyzing the percentage of EH in the cochlear apex of patients with MD. Signal intensity is indicated as a gray value, and SIRs in the phantom samples were calculated using physiological saline as the reference. In patients, potential tumor appearance in the cerebellopontine angle was screened on MRI obtained using the T_2_W-SPACE sequence. The accuracy, specificity, and sensitivity for noncontrast MRI in detecting VS were reported to reach 97%–99%, 96%–98%, and 100%, respectively [29]. T_2_W-SPACE is superior to other sequences due to the higher resolution. SIRs in the defined ROIs in the cochlear basal turn, apex and vestibule were calculated using CSF as references. In addition to identifying EH in the cochlear apex, cochlear and vestibular EH were graded according to a previous report that applied a 3-stage method for cochlear hydrops and a 4-stage scale for vestibular hydrops based on morphological changes as follows. In the cochlea, 0 = no hydrops, the interscalar septum, the scala tympani, and scala vestibuli are recognizable with the scala media normally minimally visible; I = mild hydrops, the scala media becomes a nodular black area with narrow scala vestibuli; and II = marked hydrops, the scala vestibuli becomes completely invisible. In the vestibule, 0 = no hydrops, the smaller saccule and larger utricle are visibly separated and occupy less than half of the surface of the vestibule; I = mild hydrops, the saccule becomes equal to or larger than the utricle but is not yet merged with the utricle; II = marked hydrops, the saccule merges with the utricle, and the surrounding perilymphatic space appears as a bright peripheral rim; and III = extreme hydrops, the perilymphatic enhancement of the vestibule is no longer visible [30].

### Statistical analysis

IBM SPSS 27 (IBM, Chicago, IL, USA) software was used for performing the statistical analysis. The mean values of SIRs of repeated serially diluted Gd–DTPA phantom samples imaged using hT_2_W–FLAIR–MZFI and MIIRMR were used for the statistical analysis. Univariate analysis of the general linear model and Pearson’s correlation test were applied to analyze correlations between SIR and Gd–DTPA concentrations. A paired-sample *t*-test was used for comparisons of SIR obtained with hT_2_W–FLAIR–MZFI and MIIRMR at various concentrations of Gd–DTPA in phantom samples. Student’s *t* test was applied to compare SIR differences between the cochlear basal turn and the apex and between the vestibule and the apex. A paired-sample *t* test was used for comparison of SIR obtained with hT_2_W–FLAIR–MZFI and MIIRMR in defined ROIs in the inner ear of 10 patients imaged using both hT_2_W–FLAIR–MZFI and MIIRMR. *p* < 0.05 was considered statistically significant.

## Results

### Experimental optimization of the imaging method

#### Comparing the effects of hT_2_W–FLAIR–MZFI and MIIRMR in a phantom study

**Fig. 1:**
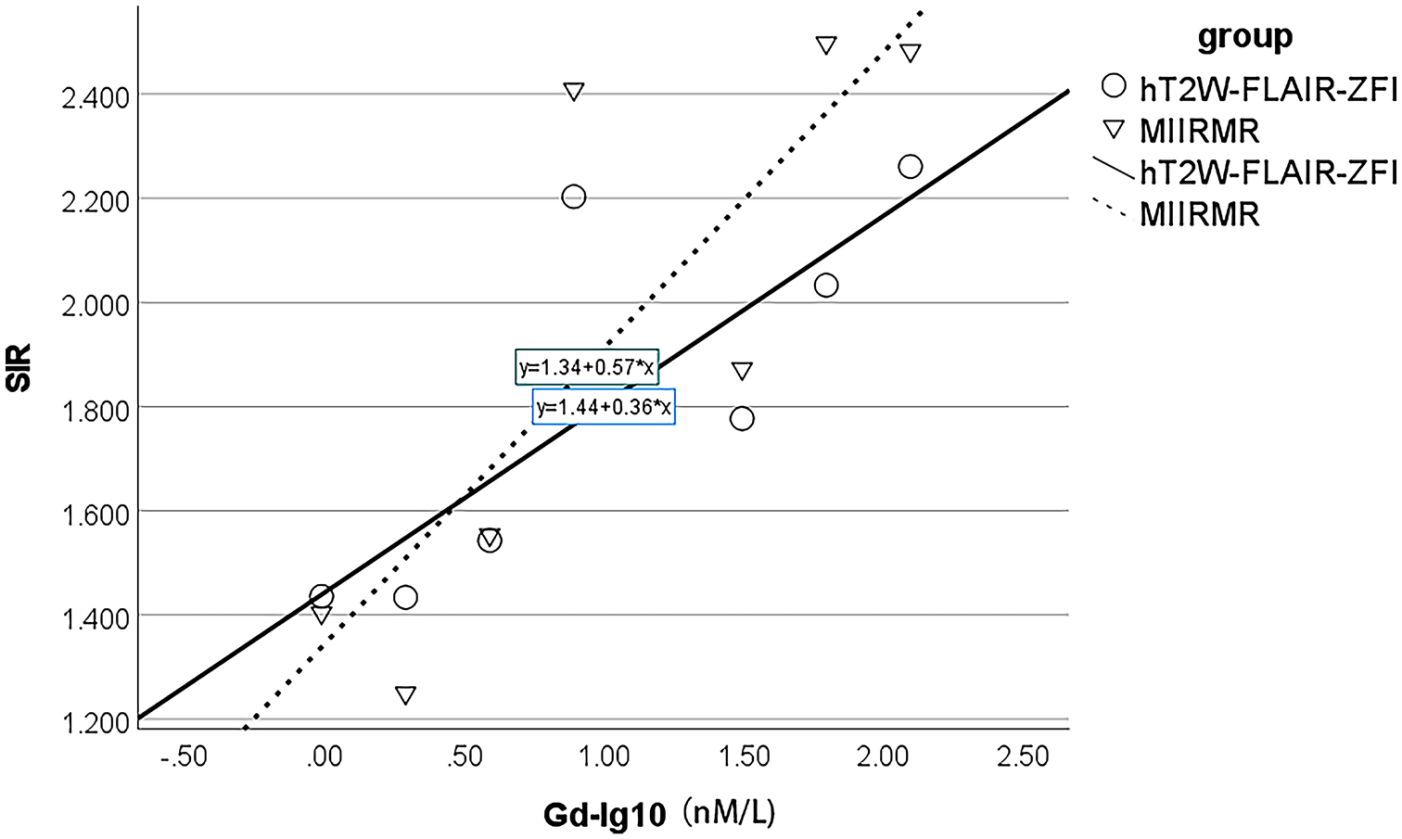
Correlations between the signal intensity ratio (SIR) and logarithm of gadolinium concentration (Gd-Log10) imaged with either hT_2_W–FLAIR–MZFI or MIIRMR in a phantom study. The slope of the curve was smaller when imaged with hT_2_W–FLAIR–MZFI than MIIRMR (0.36 versus 0.57). *R*^2^ = 0.649 (hT_2_W–FLAIR–MZFI) and 0.7 (MIIRMR)

**Fig. 2:**
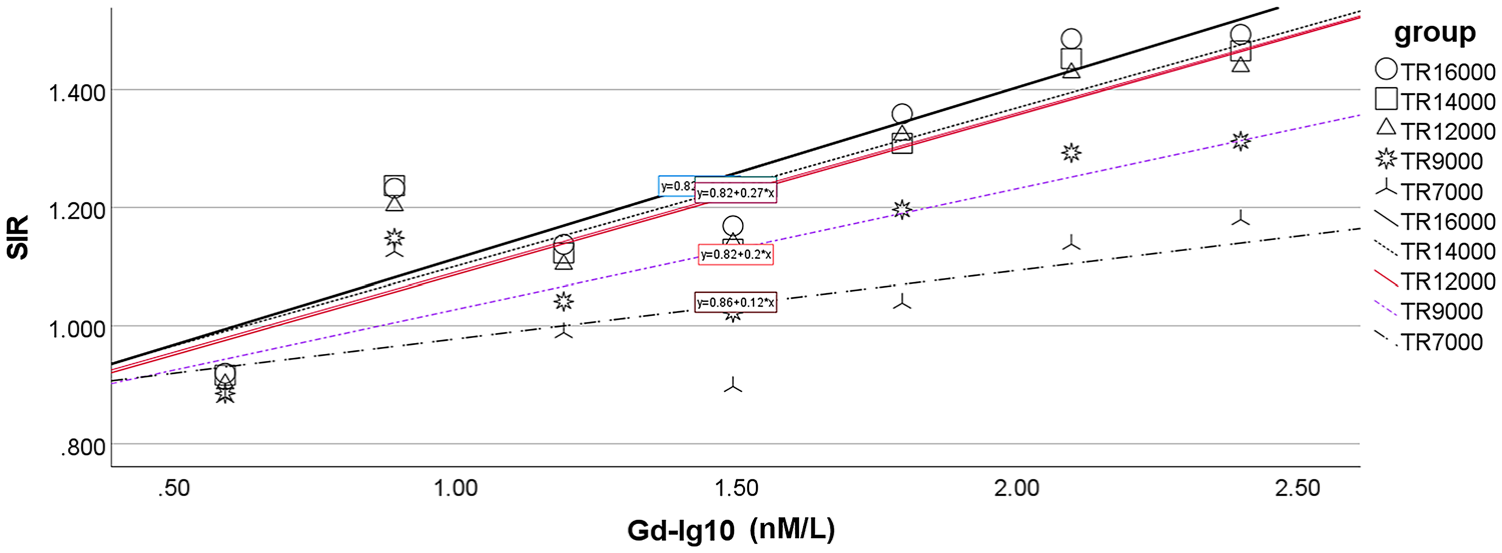
Correlations between the signal intensity ratio (SIR) and logarithm of gadolinium concentration (Gd-Log10) imaged with hT_2_W–FLAIR–MZFI at various TRs in a phantom study. Similar curves were presented by MRI with TRs ranging from 12,000 to 16,000 ms

In a phantom study, both the hT_2_W–FLAIR–MZFI and MIIRMR techniques demonstrated significant linear correlations between the SIR and logarithm of gadolinium concentration (*p* = 0.029 and 0.019, respectively). However, the curve created with hT_2_W–FLAIR–MZFI had a larger intercept and smaller slope than that created with MIIRMR (*y* = 1.44 + 0.36 *x* and* y* = 1.34 + 0.57 *x*, respectively) (Fig. 1). Equal SIR (cross point) was obtained using both hT_2_W–FLAIR–MZFI and MIIRMR at 2.99 nM Gd–DTPA. A comparison between the sensitivity and repetition time (TR) shows that the longer the TR was, the more sensitive the detection. The image sensitivity became nearly saturated when TR was longer than 12,000 ms (the curves almost overlapped from TR 12,000 to 16,000 ms) (Fig. 2).

#### Comparing the effects of MRI with hT_2_W–FLAIR–MZFI and MIIRMR in patients

**Fig. 3:**
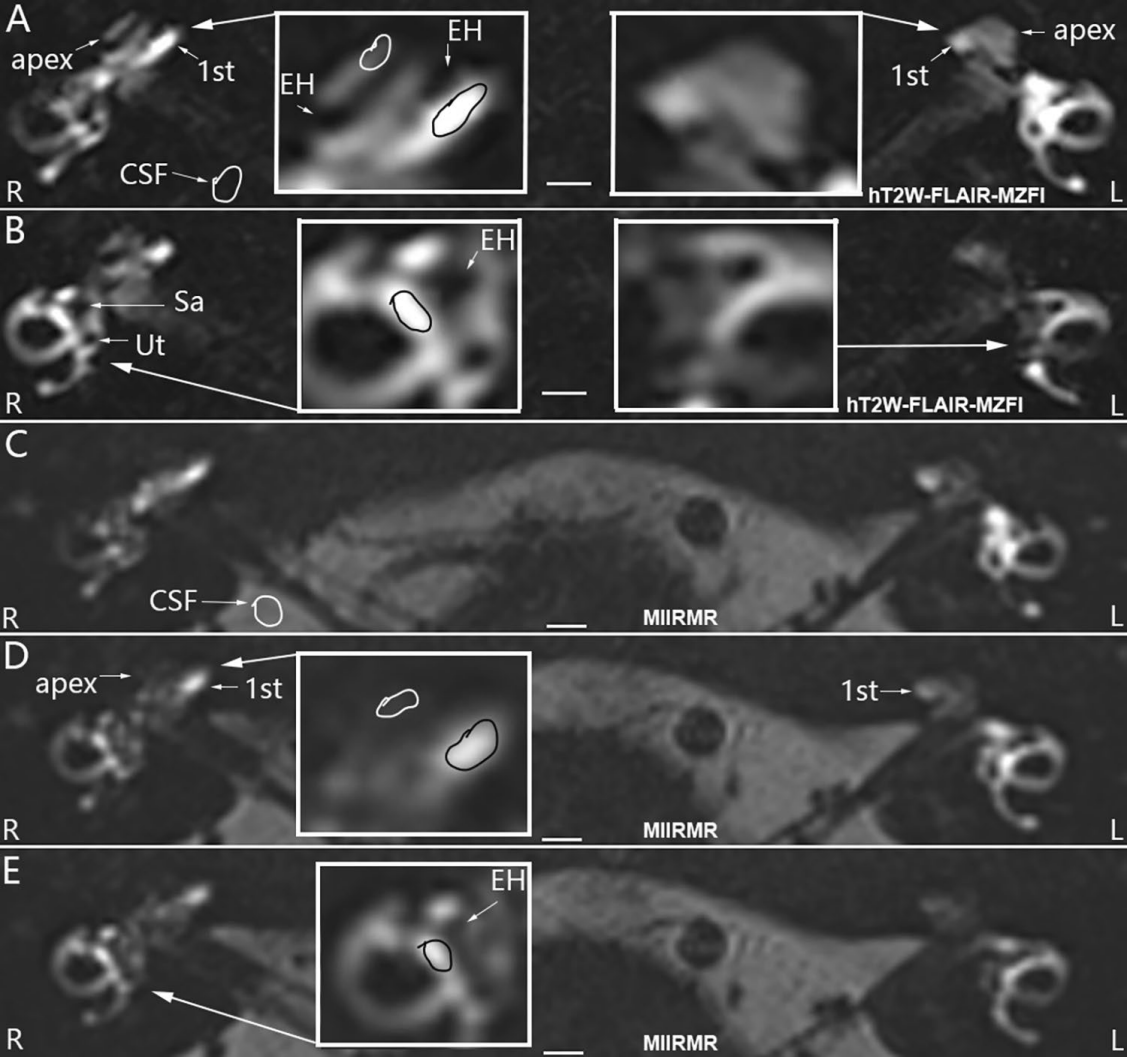
Comparison of hT_2_W–FLAIR–MZFI and MIIRMR 24 h after targeted delivery of 20-fold-diluted Gd–DTPA in a patient with Meniere’s disease. The patient suffered from episodic vertigo for 20 years and fluctuating right ear hearing loss for 1 year. The image quality and enhancement in the cochlear apex were significantly higher on MRI obtained with hT_2_W–FLAIR–MZFI **A**, **B** than on MRI obtained with MIIRMR **C**–**E**. The images acquired using MIRMR were obviously inhomogeneous **C**–**E**. Grade II cochlear endolymphatic hydrops (EH) was detected on the right (R) (zoom in **A**) using hT_2_W–FLAIR–MZFI but not with MIIRMR (zoom in **D**). Grade I vestibular EH was demonstrated in the right ear using both hT_2_W–FLAIR–MZFI (zoomed in **B**) and MIIRMR (zoomed in **E**). EH was not demonstrated in the left ear (L) imaged with either hT_2_W–FLAIR–MZFI (zoomed in **A**, **B**) or MIIRMR. Signal intensities in the regions of interest (circled areas in **A**, **B**, **D**, and **E**) were evaluated on a single slide for statistical analysis. *1st* basal turn, *CSF* cerebrospinal fluid, *Sa* saccule, *Ut* utricle. Scale bars = 3.0 mm

The inner ear MRI obtained using hT_2_W–FLAIR–MZFI displayed high-quality images with sharper and smoother borders between the endolymph and perilymph than those obtained using MIIRMR (Fig. 3). Each turn of the cochlea showed excellent contrast, including both the hydropic and normal ears without oversaturation of the enhancement signal in either the cochlear basal turn or the vestibule imaged using hT_2_W–FLAIR–MZFI (Fig. 3**A**, **B**). The apex of the diseased cochlea was vague due to the faint contrast on the images obtained using MIIRMR (Fig. 3**C**–**E**). Quantifications demonstrated that the SIRs in the cochlear basal turn, apex and vestibule imaged using hT_2_W–FLAIR–MZFI were significantly higher than those obtained with MIIRMR in patients administered Gd–DTPA with the present method. However, the SIRs in the cochlear basal turn over the apex imaged using hT_2_W–FLAIR–MZFI were significantly lower than those obtained with MIIRMR (Table 2). In other words, the hT_2_W–FLAIR–MZFI sequence detected a broader range of Gd–DTPA signals and could display an enhancement of the inner ear with less diversity than MIIRMR.

**Table 2:** Comparison of the signal intensity ratio in inner ear MRI obtained using hT_2_W–FLAIR–MZFI and MIIRMR

Sequences	SIR (mean ± SD)/*n*				
	Pairs^a^	C-BT^b^	C-Ap^b^	Vest^b^	C-BT/Ap
hT_2_W–FLAIR–MZFI	9.076 ± 5.011*/60	9.527 ± 3.519**/44	4.238 ± 1.888/44	11.553 ± 4.370**^#^/44	2.365 ± 0.703***/44
MIIRMR	1.634 ± 1.203/60	1.929 ± 0.938**/21	0.538 ± 0.435/21	2.515 ± 1.254/**21	4.073 ± 1.966/21

*C-Ap* cochlear apex, *C-BT* cochlear basal turn, *C-BT/Ap* signal intensity ratio of cochlear basal turn over apex, *hT*_*2*_*W–FLAIR–MZFI* heavily T_2_-weighted 3D fluid attenuated inversion recovery reconstructed with magnitude and zero-filled interpolation, *MIIRMR* medium inversion time inversion recovery imaging with magnitude reconstruction, *n* number of sites including cochlear basal turn, apex, and vestibule, *Vest* vestibule.

*p < 0.01 (paired *t* test comparing to those were imaged using MIIRMR)

**p < 0.01 (*t* test comparing to cochlear apex)

***p < 0.01 (*t* test comparing to those were imaged using MIIRMR)

^#^*p* < 0.05 (*t* test comparing to cochlear basal turn)

^a^Signal intensity ratio in C-Ap, C-BT- and Vest of 10 cases who were imaged with both sequences were counted

^b^Signal intensity ratio of the defined sites over the cerebrospinal fluids

### Clinical evaluation of the developed imaging method

**Fig. 4:**
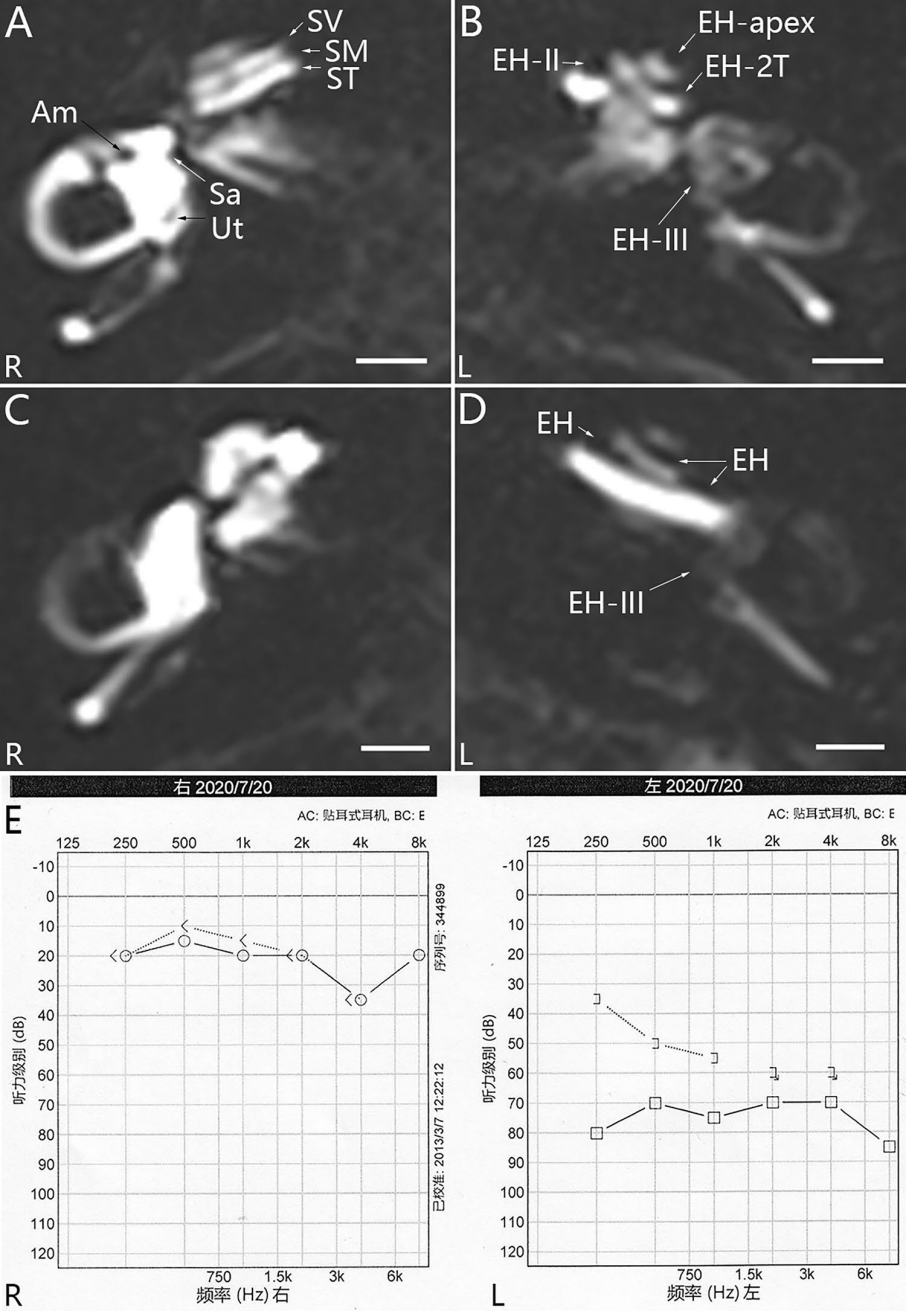
MRI of a patient with unilateral definite Meniere’s disease imaged using hT_2_W–FLAIR–MZFI 24 h after targeted delivery of 20-fold-diluted Gd–DTPA. A 49-year-old male had a history of over 8 years of episodic vertigo lasting for 5 h accompanied by fluctuating right ear hearing loss, tinnitus and aural fullness. The endolymphatic hydrops (EH) levels were grade II in the left (L) cochlea **B** and grade III in the L vestibule **D**, but EH was absent in the right ear **A**, **C**. EH was obvious in the left apex (EH-apex), which formed an invaginated arc and second turn (EH-2 T) **B**. There was severe hearing loss and a large air–bone gap at low frequencies in the left ear **E**. *Am* ampule, *Sa* saccule, *SM* scala media, *ST* scala tympani, *SV* scala vestibule, *Ut* utricle. Scale bars = 3.0 mm

**Fig. 5:**
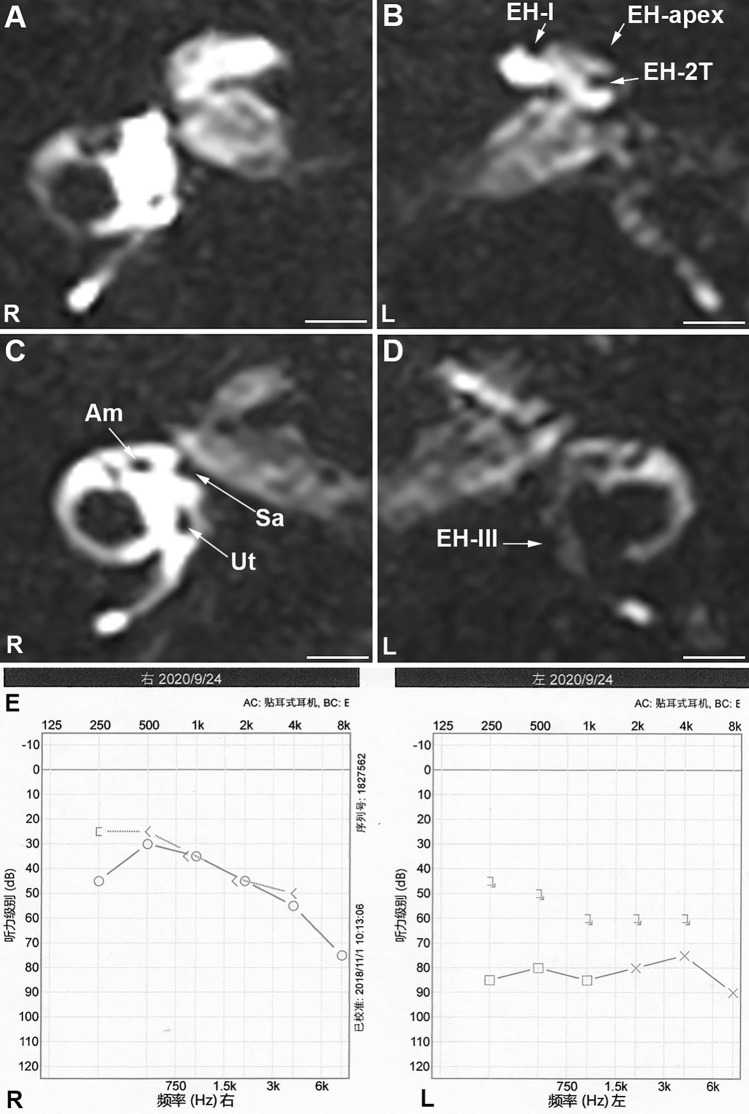
MRI of a patient with bilateral Meniere’s disease imaged using hT_2_W–FLAIR–MZFI 24 h after targeted delivery of 20-fold-diluted Gd–DTPA. A 57-year-old female had a history of over 12 years of episodic vertigo lasting for 3 h accompanied by fluctuating left ear hearing loss and bilateral tinnitus and aural fullness. The diagnosis was definite Meniere’s disease on the left side and probable Meniere’s disease on the right side. The endolymphatic hydrops (EH) was grade I in the left (L) cochlea **B** and grade III in the L vestibule **D**. EH was also detected in the L apex (EH-apex) that formed an invaginated arc and second turn (EH-2 T) **B**. No EH was detected in the right (R) ear **A**, **C**. There was profound hearing loss in the left ear and age-related hearing loss in the right ear **E**. *Am* ampule, *Sa* saccule, *Ut* utricle. Scale bars = 3.0 mm

Grading the EH in both the cochlea and the vestibule was carried out based on images acquired using hT_2_W–FLAIR–MZFI 24 h after administration of 0.1 ml of 20-fold-diluted Gd–DTPA into the middle ear cavity (Figs. 3, 4, and 5). The grades were predominantly grade II in the cochleae (11/16 ears in unilateral and 5/6 ears in bilateral) and grade III in the vestibule (8/16 ears in unilateral and 4/6 ears in bilateral) in definite MD. EH was detected in the apex of 11/16 ipsilateral ears, 3/16 contralateral ears in unilateral definite MD and 3/6 ears in bilateral MD (Table 3).

**Table 3:** Endolymphatic hydrops detected by MRI with hT_2_W–FLAIR–MZFI in patients with MD (graded 0–III and apex)

Diagnosis	*n*	Cochlear EH								Vestibular EH							
		Ipsilateral ear^a^				Contra ear^b^				Ipsilateral ear^a^				Contra ear^b^			
		0	I	II	Apex	0	I	II	Apex	0	I	II	III	0	I	II	III
Definite MD-U	16	1	4	11	11	0	0	0	3	2	2	4	8	0	0	0	0
Definite MD-B	3	1	0	2	1	0	0	3	2	0	1	0	2	1	0	0	2
Probable MD-U	2	0	0	0	0	0	0	0	0	0	0	0	0	0	0	0	0
Probable MD-B	1	0	0	0	0	0	0	0	0	0	0	0	0	0	0	0	0
SSNHL	1	0	1	0	0	0	0	0	0	0	0	0	0	0	0	0	0

*Contra* contralateral, *Definite MD-B* bilateral definite Meniere’s disease (MD), *Definite MD-U* unilateral definite MD, *EH* endolymphatic hydrops, *n* number of cases, *Probable MD-B* bilateral Probable MD, *Probable MD-U* unilateral probable MD

^a^Left ear in bilateral disease

^b^Right ear in bilateral disease

## Discussion

The present phantom study demonstrated that hT_2_W–FLAIR–MZFI is more sensitive in detecting the signal of extremely low-concentration Gd–DTPA and suppressing the signal of high-concentration Gd–DTPA than MIIRMR. The SIRs in the cochlear basal turn over the apex imaged using hT_2_W–FLAIR–MZFI were significantly lower than those obtained with MIIRMR, indicating that the hT_2_W–FLAIR–MZFI sequence yielded a broader range of Gd–DTPA signals and could display an enhancement of the inner ear with less diversity than MIIRMR. The image quality obtained with hT_2_W–FLAIR–MZFI was significantly higher than that obtained with MIIRMR in the same inner ear, presenting borders between the endolymph and perilymph that were sharper and smoother when using hT_2_W–FLAIR–MZFI than when using MIIRMR. The voxel size of hT2W–FLAIR–MZFI was significantly smaller than that of MIIRMR, which supported the advantages of hT2W–FLAIR–MZFI over MIIRMR (0.26 × 0.26 × 1.0 mm^3^ v.s. 0.52 × 0.52 × 1.0 mm^3^). The optimized parameters were also validated. Clinical MRI with the novel hT_2_W–FLAIR–MZFI technique displayed a good enhancement signal in the human inner ear, corresponding to a broad range of gadolinium concentrations, and was capable of reliably demonstrating visible enhancement in the cochlear apex. EH was frequently detected in the cochlear apex of patients with MD in addition to the lower turns and vestibule.

### Comparison of the current technique with reports from the literature

In an early clinical MRI study using the 3D-FLAIR technique, uptake of gadolinium in the inner ear was absent in 5% of ears and insufficient to evaluate EH in 13% of ears, although a significantly higher dose of gadolinium chelates than that in the present study was injected into the middle ear cavity (eightfold dilution, 0.4–0.5 ml/ear) [10]. The same group reported that a more sensitive sequence of hT_2_W–3D–FLAIR was capable of detecting gadolinium signals in the perilymph of MD patients using a 32-channel coil after intravenous injection of single-dose GdC [7, 31]. The optimized parameters were TR 9000 ms and TI 2400 ms with an imaging time of 10.7 min, which is significantly longer than that for the present study, although a head coil with many more channels than that in our study was used. However, the image quality and contrast effect were lower than those in the present study. To improve the contrast effect, Naganawa et al. developed a technique called HYDROPS (HYbriD of reversed image of positive endolymph signal and native image of positive perilymph signal) using an image calculation that was finally updated to HYDROPS-Mi2 [16]. However, the procedure is relatively complicated in clinical praxis, demands special skills in the evaluation of EH and is time-consuming (~ 18 min). Recently, Ohashi et al. simplified the technique by prolonging the repetition time and increasing the refocusing flip angle in 3D-real inversion recovery imaging, which resulted in a higher contrast-to-noise ratio than the previous sequence without image calculation and was capable of detecting EH in 11.4 min [14]. However, the contrast effect could be improved. The current hT_2_W–FLAIR–MZFI technique displayed an improved contrast effect in the inner ear from the apex to the other parts with good image quality in 7.4 min using a 3 T MR system equipped with a head coil of only 20 channels instead of 32 channels.

### Methods to improve image quality and contrast effect in the cochlear apex with hT_2_W–FLAIR–MZFI

The inner ear MRI obtained using hT_2_W–FLAIR–MZFI displayed high-quality images with sharp and smooth borders between the scalae and good contrast enhancement in the cochlear apex, where the distribution of Gd–DTPA is usually extremely sparse while avoiding overexposure artifacts in the cochlear basal turn and vestibule where the accumulation of Gd–DTPA is significantly more pronounced than the apex. The phantom study showed that the enhancement signal generated by hT_2_W–FLAIR–MZFI responding to Gd–DTPA followed a curve with a smaller slope than that generated by MIIRMR. A previous study reported that signal intensity positively correlates with the TI [32]. The longer TI in the hT_2_W–FLAIR–MZFI sequence than that in the MIIRMR sequence possibly contributes to the higher sensitivity in detecting low concentrations of Gd–DTPA. All previously reported sequences provided weak enhancement in the cochlear apex due to the partial volume effect since the apex is small relative to the nominal pixel size; moreover, there is a loss of contrast between the target and the surrounding structures [7, 17, 18, 33]. The partial volume effect reduces the contrast and continuity of the cochlear apex and the edge between the enhanced perilymph and unenhanced endolymph. In MRI using hT_2_W–FLAIR–MZFI, the data matrix size (reconstruction pixel grid) was increased in all orthogonal directions, and the unknown values were then zero-filled, which resulted in any given structure being located near a reconstructed pixel’s center, thus improving the overall visualization of the enhancement in the cochlear apex while remaining genuine to the information of the raw images [19]. The unenhanced CSF and endolymph in MRI with hT_2_W–FLAIR–MZFI were demonstrated as dark signals, possibly attributed to the longer TI than that in the previously reported MIIRMR [18], which further improves the image contrast and additionally contributes to the sharp border. A sharp border between the enhanced and unenhanced structures is beneficial for grading the EH of patients. Attributed to the greater sensitivity, enhancement of the cochlear apex was reliably detected using the hT_2_W-FLAIR-MZFI sequence, avoiding false-positive or -negative reports on EH in MD.

### Methods to reduce imaging time with hT_2_W–FLAIR–MZFI

The scanning time using hT_2_W–FLAIR–MZFI is shorter than that in previous methods. In previous reports, the imaging time was reduced by deducting slices per slab [18, 24, 25]. In the present study, the imaging time was further reduced by decreasing the TR. Although the TR in the hT_2_W–FLAIR–MZFI technique was shorter than that in the MIIRMR technique, it reached the saturation stage and did not affect the signal intensity. By decreasing the slices per slab, the procedure seems to decrease the signal-to-noise ratio, but no disadvantage was observed in practice, possibly attributed to the excellent contrast effect of the present protocol.

### The Gibbs phenomenon

In addition, known as truncation artifacts, the Gibbs phenomenon is a common image defect in MR images and appears as parallel lines or spurious rings around sharp edges or tissue discontinuities, especially in situations of a high contrast effect [34, 35]. When reviewing the images in the literature, the Gibbs phenomenon definitely occurred in MRI studies of EH using previously reported techniques, although it was not described [36–38]. This drawback was also observed in the present study. In T_2_W-SPACE images, the Gibbs phenomenon appeared as dark dots surrounding the bright fluids of the inner ear, while in hT_2_W–FLAIR–MZFI images, it occurred as parallel lines distributed below the basal turn, lateral to the cochlea, around the semicircular canals, and occasionally above the apex (Supplementary Information 3). However, the Gibbs phenomenon never existed between the cochlear scalae and within the vestibular area and did not disturb the judgment of EH. It was recently reported that the Gibbs phenomenon may be simply, effectively, and robustly removed with a minimal amount of image smoothing by reinterpolating the image based on local, subvoxel shifts to sample the ringing pattern at the zero crossings of the oscillating sinc function [39].

### Indicated clinical significance of hT_2_W–FLAIR–MZFI for EH imaging

The specificity of detecting EH using MRI was impaired by reports on EH in healthy volunteers [40]. Although the criteria for judgment of EH may be improved, a blurred border between the positive perilymph and negative endolymph may also contribute to the errors. A sharp border displayed by MRI using the hT_2_W–FLAIR–MZFI technique may improve the accuracy in EH detection. Improved visualization of the enhancement signal in the cochlear apex provides an excellent tool for thorough evaluation of EH in MD. Theoretically, the novel method may be applied in MRI with intravenous administration of GdC, which is favored by the radiologist [40].

## Study limitations

Only 23 patients were evaluated for EH using the hT_2_W–FLAIR–MZFI technique. Although all cases displayed visible enhancement in the cochlear apex, a large-sample study should be conducted in the future to further confirm the present results. A large-sample study should also be carried out to compare the accuracies of the hT_2_W–FLAIR–MZFI and MIIRMR techniques in detecting EH. The Gibbs phenomenon does exist in the present imaging protocol, although it does not disturb the judgment of EH and may be further improved with proper techniques in the future [39].

## Conclusions

The present study revealed that the novel hT_2_W–FLAIR–MZFI technique is capable of demonstrating strong enhancement by minimum Gd–DTPA in the inner ear, including the apex, and could homogenously display enhancement of the inner ear while improving the borders between enhanced and unenhanced structures. In addition, the imaging time of the present sequence is shorter than that of previously reported methods. The excellent imaging results provided an opportunity to accurately diagnose EH in the clinic.

## Supplementary Information

Below is the link to the electronic supplementary material.

Supplementary file1 (DOCX 152 KB)

## References

[1] Zou J, Pyykko I, Bjelke B, Bretlau P, and Tayamaga T (2000) Endolympahtic hydrops is caused by increased porosity of stria vascularis? Barany Society Meeting, Uppsala

[2] Zou J, Pyykko I, Bretlau P, Klason T, Bjelke B (2003). In vivo visualization of endolymphatic hydrops in guinea pigs: magnetic resonance imaging evaluation at 4.7 tesla. Ann Otol Rhinol Laryngol.

[3] Nakashima T, Naganawa S, Sugiura M, Teranishi M, Sone M, Hayashi H, Nakata S, Katayama N, Ishida IM (2007). Visualization of endolymphatic hydrops in patients with Meniere's disease. Laryngoscope.

[4] Pyykko I, Nakashima T, Yoshida T, Zou J, Naganawa S (2013). Meniere's disease: a reappraisal supported by a variable latency of symptoms and the MRI visualisation of endolymphatic hydrops. J BMJ Open.

[5] Fukushima M, Akahani S, Inohara H, Takeda N (2019). Stability of endolymphatic hydrops in Meniere disease shown by 3-tesla magnetic resonance imaging during and after vertigo attacks. JAMA Otolaryngol Head Neck Surg.

[6] Gurkov R (2017). Meniere and friends: imaging and classification of hydropic ear disease. Otol Neurotol.

[7] Naganawa S, Yamazaki M, Kawai H, Bokura K, Sone M, Nakashima T (2010). Visualization of endolymphatic hydrops in Meniere's disease with single-dose intravenous gadolinium-based contrast media using heavily T(2)-weighted 3D-FLAIR. Magn Reson Med Sci.

[8] Nakashima T, Naganawa S, Teranishi M, Tagaya M, Nakata S, Sone M, Otake H, Kato K, Iwata T, Nishio N (2010). Endolymphatic hydrops revealed by intravenous gadolinium injection in patients with Meniere's disease. Acta Otolaryngol.

[9] Zou J, Pyykko I, Counter SA, Klason T, Bretlau P, Bjelke B (2003). In vivo observation of dynamic perilymph formation using 4.7 T MRI with gadolinium as a tracer. Acta Otolaryngol.

[10] Yoshioka M, Naganawa S, Sone M, Nakata S, Teranishi M, Nakashima T (2009). Individual differences in the permeability of the round window: evaluating the movement of intratympanic gadolinium into the inner ear. Otol Neurotol.

[11] Zou J, Pyykko I, Bjelke B, Dastidar P, Toppila E (2005). Communication between the perilymphatic scalae and spiral ligament visualized by in vivo MRI. Audiol Neurootol.

[12] Inui H, Sakamoto T, Ito T, Kitahara T (2019). Magnetic resonance imaging of the endolymphatic space in patients with acute low-tone sensorineural hearing loss. Auris Nasus Larynx.

[13] Nakashima T, Naganawa S, Pyykko I, Gibson WP, Sone M, Nakata S, Teranishi M (2009). Grading of endolymphatic hydrops using magnetic resonance imaging. Acta Otolaryngol Suppl.

[14] Ohashi T, Naganawa S, Takeuchi A, Katagiri T, Kuno K (2020). Quantification of endolymphatic space volume after intravenous administration of a single dose of gadolinium-based contrast agent: 3D-real inversion recovery versus HYDROPS-Mi2. Magn Reson Med Sci.

[15] Dunnebier EA, Segenhout JM, Wit HP, Albers FW (1997). Two-phase endolymphatic hydrops: a new dynamic guinea pig model. Acta Otolaryngol.

[16] Naganawa S, Suzuki K, Nakamichi R, Bokura K, Yoshida T, Sone M, Homann G, Nakashima T, Ikeda M (2013). Semi-quantification of endolymphatic size on MR imaging after intravenous injection of single-dose gadodiamide: comparison between two types of processing strategies. Magn Reson Med Sci.

[17] Naganawa S, Kawai H, Taoka T, Sone M (2019). Improved 3D-real inversion recovery: a robust imaging technique for endolymphatic hydrops after intravenous administration of gadolinium. Magn Reson Med Sci.

[18] Zou J, Wang Z, Chen Y, Zhang G, Chen L, Lu J (2019). MRI detection of endolymphatic hydrops in Meniere's disease in 8 minutes using MIIRMR and a 20-channel coil after targeted gadolinium delivery. World J Otorhinolaryngol Head Neck Surg.

[19] Elgavish RA, Twieg DB (2003). Improved depiction of small anatomic structures in MR images using Gaussian-weighted spirals and zero-filled interpolation. Magn Reson Imaging.

[20] Du YP, Parker DL, Davis WL, Cao G (1994). Reduction of partial-volume artifacts with zero-filled interpolation in three-dimensional MR angiography. J Magn Reson Imaging.

[21] Forbes KP, Pipe JG, Karis JP, Heiserman JE (2003). Effects of zero-filled interpolation on cervical magnetic resonance angiographic measurement. AJNR Am J Neuroradiol.

[22] Avci E, Nauwelaers T, Lenarz T, Hamacher V, Kral A (2014). Variations in microanatomy of the human cochlea. J Comp Neurol.

[23] Walby AP (1985). Scala tympani measurement. Ann Otol Rhinol Laryngol.

[24] Zou J, Wang Z, Chen Y, Zhang G, Lu J, Zheng H (2018). Detecting endolymphatic hydrops with posterior tympanic medial wall Gd-DTPA delivery and 8 min-MRI.

[25] Zou J, Wang Z, Chen YK, Zhang GP, Lu JP, Zheng HL (2018). Optimization of delivering minimum Gd-DTPA at the posterior upper point on tympanic medial wall and hT2W-3D-FLAIR sequence for detecting endolymphatic hydrops. Zhonghua Er Bi Yan Hou Tou Jing Wai Ke Za Zhi.

[26] Thalmann I, Comegys TH, Liu SZ, Ito Z, Thalmann R (1992). Protein profiles of perilymph and endolymph of the guinea pig. Hear Res.

[27] Lopez-Escamez JA, Carey J, Chung WH, Goebel JA, Magnusson M, Mandala M, Newman-Toker DE, Strupp M, Suzuki M, Trabalzini F, Bisdorff A, Classification Committee of the Barany S, Japan Society for Equilibrium R, European Academy of O, Neurotology, Equilibrium Committee of the American Academy of O-H, Neck S, and Korean Balance S (2015). Diagnostic criteria for Meniere's disease. J Vestib Res.

[28] Stachler RJ, Chandrasekhar SS, Archer SM, Rosenfeld RM, Schwartz SR, Barrs DM, Brown SR, Fife TD, Ford P, Ganiats TG, Hollingsworth DB, Lewandowski CA, Montano JJ, Saunders JE, Tucci DL, Valente M, Warren BE, Yaremchuk KL, Robertson PJ, American Academy of O-H and Neck S (2012). Clinical practice guideline: sudden hearing loss. Otolaryngol Head Neck Surg.

[29] Zou J, Hirvonen T (2017). "Wait and scan" management of patients with vestibular schwannoma and the relevance of non-contrast MRI in the follow-up. J Otol.

[30] Bernaerts A, Vanspauwen R, Blaivie C, van Dinther J, Zarowski A, Wuyts FL, Vanden Bossche S, Offeciers E, Casselman JW, De Foer B (2019). The value of four stage vestibular hydrops grading and asymmetric perilymphatic enhancement in the diagnosis of Meniere's disease on MRI. Neuroradiology.

[31] Naganawa S, Kawai H, Sone M, Nakashima T (2010). Increased sensitivity to low concentration gadolinium contrast by optimized heavily T2-weighted 3D-FLAIR to visualize endolymphatic space. Magn Reson Med Sci.

[32] Shen Y, Goerner FL, Snyder C, Morelli JN, Hao D, Hu D, Li X, Runge VM (2015). T1 relaxivities of gadolinium-based magnetic resonance contrast agents in human whole blood at 1.5, 3, and 7 T. Invest Radiol.

[33] Naganawa S, Yamazaki M, Kawai H, Bokura K, Iida T, Sone M, Nakashima T (2014). MR imaging of Meniere's disease after combined intratympanic and intravenous injection of gadolinium using HYDROPS2. Magn Reson Med Sci.

[34] Czervionke LF, Czervionke JM, Daniels DL, Haughton VM (1988). Characteristic features of MR truncation artifacts. AJR.

[35] Lufkin RB, Pusey E, Stark DD, Brown R, Leikind B, Hanafee WN (1986). Boundary artifact due to truncation errors in MR imaging. AJR Am J Roentgenol.

[36] Naganawa S, Satake H, Kawamura M, Fukatsu H, Sone M, Nakashima T (2008). Separate visualization of endolymphatic space, perilymphatic space and bone by a single pulse sequence; 3D-inversion recovery imaging utilizing real reconstruction after intratympanic Gd-DTPA administration at 3 Tesla. Eur Radiol.

[37] Shi H, Li Y, Yin S, Zou J (2014). The predominant vestibular uptake of gadolinium through the oval window pathway is compromised by endolymphatic hydrops in Meniere's disease. Otol Neurotol.

[38] Wu Q, Dai C, Zhao M, Sha Y (2016). The correlation between symptoms of definite Meniere's disease and endolymphatic hydrops visualized by magnetic resonance imaging. Laryngoscope.

[39] Kellner E, Dhital B, Kiselev VG, Reisert M (2016). Gibbs-ringing artifact removal based on local subvoxel-shifts. Magn Reson Med.

[40] Conte G, Lo Russo FM, Calloni SF, Sina C, Barozzi S, Di Berardino F, Scola E, Palumbo G, Zanetti D, Triulzi FM (2018). MR imaging of endolymphatic hydrops in Ménière’s disease: not all that glitters is gold. Acta Otorhinolaryngol Ital.

