# Correction to: High-quality imaging of endolymphatic hydrops acquired in 7 minutes using sensitive hT_2_W–3D–FLAIR reconstructed with magnitude and zero-filled interpolation

**DOI:** 10.1007/s00405-021-07216-3

**Published:** 2021-12-18

**Authors:** Jing Zou, Luguang Chen, Hongbin Li, Guoping Zhang, Ilmari Pyykkö, Jianping Lu

**Affiliations:** 1 Department of Otolaryngology-Head and Neck Surgery, Center for Otolaryngology-Head and Neck Surgery of Chinese PLA, Changhai Hospital, Second Military Medical University, Shanghai, China; 2 Department of Radiology, Changhai Hospital, Second Military Medical University, Shanghai, China; 3 Hearing and Balance Research Unit, Field of Otolaryngology, School of Medicine, Faculty of Medicine and Health Technology, Tampere University, Tampere, Finland

## Correction to: European Archives of Oto-Rhino-Laryngology 10.1007/s00405-021-06912-4

In the original publication of the article, under the legend of figure 4, “fluctuating *right* ear hearing loss” should be replaced by “fluctuating *left* ear hearing loss”. The correct legend is provided below,

**Fig. 4** MRI of a patient with unilateral definite Meniere’s disease imaged using hT2W-FLAIR-MZFI 24 h after targeted delivery of 20-fold-diluted Gd-DTPA. A 49-year-old male had a history of over 8 years of episodic vertigo lasting for 5 h accompanied by fluctuating left ear hearing loss, tinnitus and aural fullness. The endolymphatic hydrops (EH) levels were grade II in the left (L) cochlea **B** and grade III in the L vestibule (**D**), but EH was absent in the right ear **A**, **C**. EH was obvious in the left apex (EH-apex), which formed an invaginated arc and second turn (EH-2 T) **B**. There was severe hearing loss and a large air–bone gap at low frequencies in the left ear **E**. *Am* ampule, *Sa* saccule, *SM* scala media, *ST* scala tympani, *SV* scala vestibule, *Ut* utricle. Scale bars 3.0 mm

