# Supplementary material for: High-quality imaging of endolymphatic hydrops acquired in 7 minutes using sensitive hT2W–3D–FLAIR reconstructed with magnitude and zero-filled interpolation
Source: Eur Arch Otorhinolaryngol. 2021 Jun 18;279(5):2279–90. doi: 10.1007/s00405-021-06912-4 (PMC8986670; doi:10.1007/s00405-021-06912-4)
Supplement: Supplementary file 1 — Supplementary file1 (DOCX 152 KB) [file 405_2021_6912_MOESM1_ESM.docx]

**Supplementary Information**

**
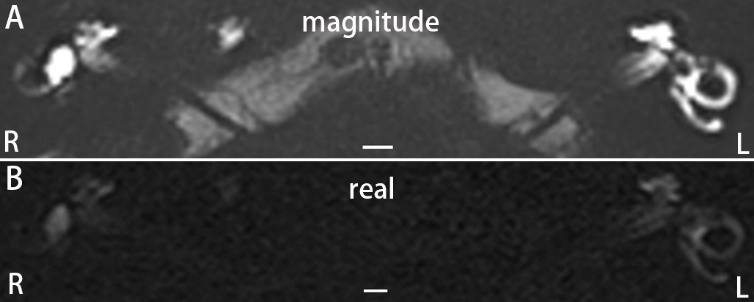
**

**Supplementary Information 1**. Comparison between magnitude reconstruction of MIIRMR and real reconstruction of the same scan 24 h after targeted intratympanic delivery of 20-fold-diluted Gd-DTPA in a patient with Meniere’s disease. The patient suffered from fluctuating bilateral hearing loss for 9 months before the MRI was performed but developed episodic vertigo after the MRI. The signal intensity in MRI with magnitude reconstruction (A) was significantly greater than that with real reconstruction (B). There were cochlear and vestibular endolymphatic hydrops in the left ear (L) and cochlear endolymphatic hydrops in the right ear (R). Scale bars = 3.0 mm.

MD (22)

SSNHL (1)

hT_2_W-FLAIR-MZFI + MIIRMR + 3D-real IR (1)

hT_2_W-FLAIR-MZFI (12)

hT_2_W-FLAIR-MZFI + MIIRMR (10)

Pilot^*^ + Comparison^#^ + EH evaluation

EH evaluation

Comparison + EH evaluation

**Supplementary Information 2**. The study flow diagram of patient distribution. *A pilot study was performed to compare the enhancement effect between magnitude reconstruction and real reconstruction of MIIRMR in the same scan. #Comparing the enhancement effect of hT_2_W-FLAIR-MZFI and MIIRMR. 3D-real IR: 3D-real inversion recovery; EH: endolymphatic hydrops; MD: Meniere’s disease; SSNHL: sudden sensorineural hearing loss.


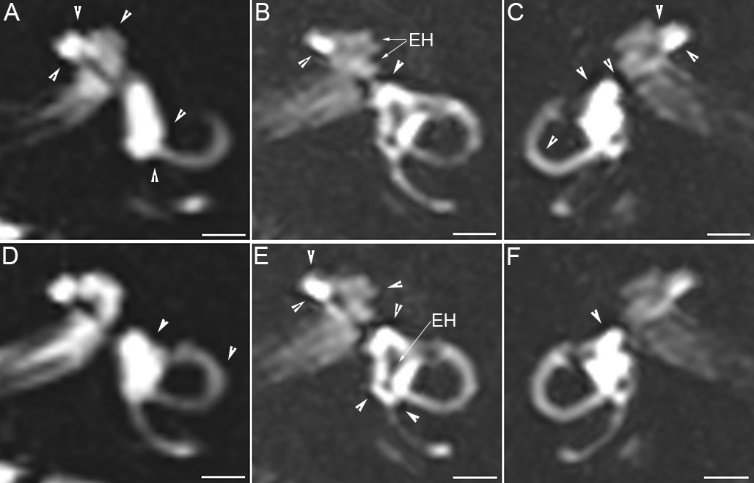


**Supplementary Information 3**. The Gibbs phenomenon in inner ear MRI. The Gibbs phenomenon (arrowheads) appeared as dots or parallel lines around the bright signals in MRI obtained with SPACE (A, D) or hT_2_W-FLAIR-MZFI (B, C, E, F). Endolymphatic hydrops (EH) in the cochlea was depicted as a dark area that extruded the cochlear scala to form an invaginated arc (B). Scale bars = 3.0 mm.
